# The dose divided

**DOI:** 10.1017/S1478951526102946

**Published:** 2026-06-10

**Authors:** Tarek Zieneldien

**Affiliations:** The Johns Hopkins University School of Medicine, Baltimore, MD, USA

Radiation comes in weekdays,
Hope measured out
In calibrated light

Every morning,
The body returns in nervous posture,
To the same unadorned white room,
To the same mask cooled against the face,
To the same machine that knows
How to divide harm
From healing so finely
They nearly share a name

She lies
While the beam is delivered, trespassing
In exactness,
As if the body could be persuaded
One small portion at a time
To remain still,
And still, remain

In the hall,
An old woman folds her appointment card
Into something like a prayer
Outside, deadly nightshade petals gather
In the gutter,
Purple as a bruise
The season refuses to explain

She thinks of Avicenna
Crossing sandstorms by memory and vow,
The road splitting his life
Into days of dust,
Each step a fraction
Of the body’s faith
He survives, his companion does not

By Friday, the chart calls it a fraction
The body calls it return
Before the weekend, you visualize the fractals
The possibilities, an elsewhere of health

